# Secreted proteins from *Bacillus subtilis* K1 induce apple resistance against gray mold

**DOI:** 10.3389/fpls.2026.1825616

**Published:** 2026-05-04

**Authors:** Peiqian Li, Yuanlin Sun, Baozhen Feng, Lili Wei

**Affiliations:** 1 Department of Life Sciences, Yuncheng University, Yuncheng, Shanxi, China; 2 Shanxi Key Laboratory of Yuncheng Salt Lake Ecological Protection and Resource Utilization, Yuncheng, Shanxi, China; 3 Shanxi Technology Innovation Center of High Value-Added Echelon Utilization of Premium Agro-Products, Yuncheng, Shanxi, China

**Keywords:** biocontrol, *Botrytis cinerea*, elicitor, enhance resistance, secreted protein

## Abstract

The antagonistic *Bacillus subtilis* K1, previously isolated in our laboratory, exhibited significant inhibitory activity against *Botrytis cinerea*. To investigate the underlying molecular mechanisms, genomic bioinformatics analysis predicted 93 potential secreted proteins. Preliminary screening via gene cloning and an Agrobacterium-mediated transient expression system in tobacco identified a gene, *GM001344*, which elicited a strong hypersensitive response on tobacco. The GM001344-His recombinant protein was successfully purified using a prokaryotic expression system. Apple fruit inoculation assays showed that pretreatment with GM001344 protein 24 h prior to *B. cinerea* challenge markedly reduced both disease incidence (63.5% vs. 100% in control) and disease severity index (38.43 vs. 76.75 in control), demonstrating effective disease control. GM001344 enhanced apple defenses through boosted activities of key defense-related enzymes and reduced malondialdehyde (MDA) content. Furthermore, GM001344 treatment improved postharvest fruit quality. After 6 d of treatment, treated fruit maintained significantly higher firmness, total soluble solid and ascorbic acid compared to the control. In conclusion, the secreted protein GM001344 from K1 exhibits elicitor activity, effectively inducing systemic resistance against gray mold in apple and delaying quality deterioration. This study provides promising candidate protein and a preliminary theoretical basis for the development of novel biopesticides based on protein elicitors.

## 1 Introduction

Apple production systems worldwide incur substantial economic losses due to postharvest decay, with fungal pathogens representing a major causative factor (Xu et al., 2025; Dai et al., 2025). Among these, *Botrytis cinerea* (Pers.), the causal agent of gray mold, is a particularly destructive postharvest pathogen (Liu et al., 2025; Yu et al., 2024). This necrotrophic fungus not only causes soft rot, leading to significant quantitative and qualitative losses throughout storage and logistic chains, but also often facilitates secondary infections by other microorganisms. Its broad host spectrum, coupled with a cold-hardiness that allows it to remain active even at common cold-storage temperatures, and its capacity to form persistent survival structures (sclerotia) collectively establish *B. cinerea* as a pervasive and recalcitrant postharvest problem, continuously threatening the sustainability and profitability of global apple production (Bi et al., 2023; Chen et al., 2023; Veloso and van Kan, 2018). Conventional management has predominantly depended on applications of synthetic fungicides. However, this strategy is increasingly limited by several critical drawbacks. Mounting public and regulatory concerns over pesticide residues on food products and their adverse environmental impacts have led to stricter regulations and heightened consumer demand for residue-free alternatives (Kaur et al., 2024; Gilbert and Edwin, 2021). Moreover, the intensive, and often repetitive, use of site-specific fungicides has exerted strong selection pressure, resulting in the widespread emergence of resistant *B. cinerea* populations, thereby eroding the efficacy of existing chemical controls (Wu et al., 2025; Leroux et al., 2002). These interrelated challenges highlight the urgent imperative to develop and implement effective, environmentally benign, and sustainable alternative strategies within modern integrated pest management (IPM) frameworks for postharvest disease suppression (Song et al., 2024; Egan et al., 2020).

The pursuit of sustainable agriculture has established biological control as a fundamental component of modern crop protection strategies. Within this paradigm, microbial biocontrol agents (BCAs) offer a compelling synergy of efficacy and ecological compatibility (Pirttilä et al., 2021). Notably, members of the genus *Bacillus* have risen to prominence as leading candidates, attracting extensive research interest owing to a combination of inherent advantages. These include exceptional environmental resilience, which ensures survival under fluctuating field conditions; a wide antagonistic range against diverse plant pathogens; and favorable traits for economical, large-scale production and formulation (Lastochkina et al., 2019). Together, these characteristics render *Bacillus* species highly adaptable and operationally feasible agents for integration into comprehensive pest management systems (Wang et al., 2022; Lastochkina et al., 2019). *Bacillus* spp. exhibit strong and broad-spectrum antagonistic capacity. This activity is primarily mediated through the secretion of a diverse array of antimicrobial metabolites, including lipopeptides, polyketides, and hydrolytic enzymes. Another significant benefit is their plant growth-promoting traits, which enhance host vigor and abiotic stress tolerance (Torres-García et al., 2024; Feng et al., 2024; Aboelez et al., 2024; Zhong et al., 2024; Chaouachi et al., 2021). Critically, *Bacillus* spp. also possess inherent safety for food and environmental applications. Many strains carry Generally Recognized As Safe (GRAS) status, alleviating key regulatory and safety concerns (Ali et al., 2025; Zhang et al., 2020).

Proteins play a vital role in postharvest disease management of fruits and vegetables, primarily by exerting direct antimicrobial effects and activating inherent host defense mechanisms. The use of antifungal proteins (AFPs), exemplified by the efficacy of PAFB in reducing *Penicillium digitatum*, *P. italicum*, and *P. expansum* infections in citrus and apple fruits, highlights their significant potential in controlling postharvest fungal decay (Gandía et al., 2021). The recombinant protein SlRP5 significantly enhances tomato resistance to *Botrytis cinerea* by activating comprehensive immune responses, including the induction of ROS, activation of MAPK pathways, promotion of callose deposition, and upregulation of genes involved in calcium-binding and phenylpropanoid biosynthesis (Man et al., 2025). Secreted proteins from various organisms, including bacteria and fungi, have been shown to trigger ISR (Induced Systemic Resistance), thereby enhancing plant immunity against a broad spectrum of pathogens. For instance, the secreted protein PgSCP from the biocontrol yeast *Pichia galeiformis* interacts with the citrus transcription factor CsMIKC, leading to the activation of defense genes and enhanced resistance against citrus green mold (Chen et al., 2025). The fungal protein PdEIX from *Penicillium digitatum* has been shown to function as an effective elicitor, inducing plant defense mechanisms and boosting disease resistance in harvested fruit (He et al., 2025). PgSLP, a secreted protein from *Pichia galeiformis*, enhances citrus resistance to blue mold by upregulating amino acid synthesis and metabolism pathways, increasing key enzyme activities and elevating the levels of defensive amino acids and glutathione (Xu et al., 2024). While *Bacillus* strains are widely studied for biocontrol, research on their secreted proteins remains limited. Emerging evidence suggests these proteins may extend beyond direct antimicrobial activity to include functions such as inducing plant systemic resistance. Further exploration of the *Bacillus* secretome is therefore needed to fully elucidate its potential in enhancing postharvest disease management.

Previous research has demonstrated the efficacy of *Bacillus subtilis* K1 against gray mold, with its culture filtrate activating defense responses in fruit, implicating secreted proteins in this process (Feng et al., 2025; Li et al., 2022). To pinpoint the key mediators, this study combined genomic bioinformatics screening with functional validation, identifying a specific secreted protein gene whose overexpression enhances apple resistance to *B. cinerea*. These findings elucidate the molecular mechanism of protein-induced systemic resistance in apple, providing both a novel target for developing biocontrol agents and a foundation for sustainable postharvest disease management strategies.

## 2 Materials and methods

### 2.1 Strains and plant materials

In this study, *Bacillus subtilis* K1 (Li et al., 2022) was regularly maintained in 30% glycerol stocks at −80 °C. K1 was routinely cultured in LB medium at 28 °C with shaking at 180 rpm. *Botrytis cinerea* strain was cultured on PDA medium at 24 °C, and conidia were utilized at the concentration of 1 × 10^6^ spores/mL. *Escherichia coli* strains DH5α and BL21(DE3) were grown in LB liquid medium at 37 °C with shaking at 220 rpm. *Agrobacterium tumefaciens* GV3101 was maintained in LB medium supplemented with 50 µg/mL rifampicin at 28 °C with shaking at 220 rpm. The Potato virus X (PVX)-based vector pgR106 was used for transient expression assays (Jones et al., 1999).

Fruit inoculation experiment used healthy ‘Fuji’ apple (*Malus pumila* Mill) with uniform ripeness and size. In preparation for the following experimental steps, apples were immersed in a 0.2% (v/v) NaOCl solution for 2 minutes, rinsed extensively with sterile water, and then allowed to air-dry.

*Nicotiana benthamiana* plants were cultivated in an artificial climate incubator at 25 °C with a 14 h day/10 h night cycle at 70% relative humidity. Plants at the 4- to 6-week-old stage were subjected to transient expression assays via *Agrobacterium*-mediated transformation.

### 2.2 Prediction analysis of secreted proteins from K1 genome

Genome sequences of strain K1 have been deposited in NCBI under accession number CP093546 (Li et al., 2022). Putative secretory proteins of *Bacillus subtilis* were identified bioinformatically. Initial screening with SignalP 6.0 (http://www.cbs.dtu.dk/services/SignalP/) identified proteins with signal peptides (Teufel et al., 2022). These candidates were analyzed with TMHMM 2.0 (http://www.cbs.dtu.dk/services/TMHMM/) to distinguish soluble secreted proteins from those potentially anchored to the membrane (Krogh et al., 2001). The latter were examined for specific cell wall motifs using ScanProsite (https://prosite.expasy.org/scanprosite/) (de Castro et al., 2006), while LipoP 1.0 (http://www.cbs.dtu.dk/services/LipoP/) identified lipoproteins (Juncker et al., 2003). Subcellular localizations were consolidated using PSORTb 3.0 (https://www.psort.org/psortb/) (Yu et al., 2010), and functional domains were annotated with InterProScan (https://www.ebi.ac.uk/interpro/search/sequence/) (Blum et al., 2021) and Blast2GO (https://www.blast2go.com/) (Conesa et al., 2005). Protein sequences were functionally annotated by assigning KEGG Orthology (KO) identifiers through the Kyoto Encyclopedia of Genes and Genomes (KEGG) database, enabling subsequent reconstruction of KEGG metabolic pathways (Mao et al., 2005).

### 2.3 Gene cloning and vector construction for transient expression

#### 2.3.1 Gene cloning of candidate genes

*Bacillus subtilis* strain K1 was incubated in LB medium under conditions of 28 °C and 180 rpm shaking until the optical density at 600 nm (OD_600_) attained 0.8. Following cell harvest, genomic DNA was isolated from K1 using a commercial bacterial DNA extraction kit (Sangon Biotech, Shanghai, China; Catalog No. B518255). The complete cDNA sequences of the candidate genes were then amplified via PCR with the primers (**Supplementary Table 1**), using the extracted genomic DNA as template. The final PCR product was validated by sequencing performed at Sunbiotech (Beijing, China).

#### 2.3.2 Potato virus X vector construction

For transient expression mediated by *Agrobacterium*, both the full-length *GM001344* (including its native signal peptide) and the truncated form *GM001344*^ΔSP^ were first amplified by PCR using primers listed in **Supplementary Table 1**. After purification, the resulting amplicons were cloned into the Potato virus X (PVX) vector pgR106 pre-digested with *Cla*I and *Sal*I. The recombinant constructs were confirmed by sequencing before being introduced into *Agrobacterium tumefaciens* GV3101 competent cells via electroporation. Briefly, 1 µL (100 ng) of plasmid DNA was added to 50 µL of electrocompetent GV3101 cells on ice. The mixture was transferred to a chilled 0.1 cm electroporation cuvette and subjected to a single pulse at 1.8 kV, 200 Ω, and 25 µF using a Gene Pulser Xcell (Bio-Rad, USA). Immediately after the pulse, 1 mL of ice-cold LB medium was added, and the cells were incubated at 28 °C for 2 h with gentle shaking. Transformants were selected on LB agar plates supplemented with 25 μg/mL rifampicin and 50 μg/mL kanamycin, followed by incubation at 28 °C for 48 hours to obtain positive clones.

#### 2.3.3 Detection of hypersensitive response in tobacco leaves

The ability of GM001344 to elicit an HR in *N. benthamiana* was assessed by Agrobacterium-mediated transient expression following the established protocols of Vergish et al. (2024) and Mo et al. (2024). A single colony *Agrobacterium tumefaciens* strain GV3101 harboring the recombinant plasmid was inoculated into 5 mL of LB medium containing 50 mg/L kanamycin and 25 mg/L rifampicin, and cultured overnight at 28 °C with shaking at 200 rpm. The next day, 1 mL of the overnight culture was transferred into 50 mL of fresh LB medium supplemented with the same antibiotics, 10 mM MES (pH 5.6), and 20 μM acetosyringone, then grown to an OD_600_ of 0.6–0.8. Cells were harvested by centrifugation (4, 000 × g, 10 min, 25 °C) and resuspended in infiltration buffer (10 mM MgCl_2_, 10 mM MES pH 5.6, 150 µM acetosyringone) to a final OD_600_ of 0.5. The suspension was incubated at room temperature for 2–3 hours before infiltration. For infiltration, fully expanded leaves of 4–6 week-old *Nicotiana benthamiana* plants were used. The bacterial suspension was injected into the abaxial side of the leaves using a needleless 1 mL syringe. After infiltration, plants were grown under controlled conditions (25 °C, 14h light/10 h dark, 70% relative humidity). Three leaves were used per treatment, and the experiment was conducted in triplicate and repeated three times.

The accumulation of reactive oxygen species (ROS) and the deposition of callose are important markers of the plant hypersensitive response. Accumulation of reactive oxygen species (ROS) was visualized using 3, 3′-diaminobenzidine (DAB) staining according to a published protocol (Kumar et al., 2023). Briefly, leaf tissue was immersed in DAB solution (1 mg/mL, pH 3.8) and kept in darkness at room temperature for 14 h. Subsequently, the samples were washed with distilled water, gently dried, and cleared by incubation in 95% ethanol at 40 °C. In DAB staining experiment, three leaves were used per treatment, and the entire experiment was independently repeated three times.

Callose deposition was assessed in tobacco leaves via aniline blue fluorescence staining, following the method described by Chalfoun et al. (2013). Leaves were first cleared in a solution of ethanol and acetic acid (3:1, v/v), rinsed twice with absolute ethanol (1 min each), and then sequentially equilibrated in 50% ethanol and 1× PBS (30 min per step). After staining with an aniline blue working solution for 1 h in the dark, samples were mounted and visualized under UV fluorescence microscopy. For aniline blue staining experiment, three leaves were used per treatment, and the entire experiment was independently repeated three times.

### 2.4 Construction of prokaryotic expression vector and protein purification

#### 2.4.1 Construction of prokaryotic expression vector

A prokaryotic expression vector was assembled via homologous recombination using the In-Fusion Snap Assembly Master Mix (Takara Bio, Dalian, China). The *GM001344* gene, lacking its signal peptide (SP), was inserted into the pET28a vector through *Hind* III and *EcoR* I restriction sites. Following sequence confirmation, the plasmid was purified and introduced into *Escherichia c*oli BL21(DE3) competent cells for further protein expression analysis. Primers used were listed in **Supplementary Table 1**.

To obtain recombinant protein, the transformants was transferred at a ratio of 5% (v/v) into LB liquid medium containing kanamycin and incubated with shaking until the OD_600_ reached 0.6–0.8. IPTG (Isopropyl β-D-1-thiogalactopyranoside) was then added to a final concentration of 0.1 mmol/L, and the culture was further incubated at 20 °C with shaking at 180 rpm for 5 h. A negative control was established using the strain *E. coli* BL21(DE3) harboring the empty pET28a vector.

#### 2.4.2 Protein purification of GM001344-His

Cell harvesting post-induction was performed by centrifugation at 6, 000 × g for 15 min at 4 °C. After a wash step with PBS buffer (0.1 mM, pH 7.4), cell lysis was achieved through ultrasonication in an ice bath using 3 s pulses with 3 s intervals for 30 min. to generate the crude protein extract. Affinity purification utilizing a BeaverBeads^®^ IDA-Nickel Kit-10 (Beaverbio, China) was employed to isolate the His-tagged target protein. The eluate was concentrated by ultrafiltration (0.22 µm, Millipore) and evaluated via SDS-PAGE. Protein concentration measurement was conducted using a Bradford assay kit, with readings taken at 595 nm on a microplate reader.

#### 2.4.3 Bioactivity of GM001344-His against *Botrytis cinerea*

100 µL of the protein solution (20 µM) were uniformly coated onto the PDA plate. After air drying, a *B. cinerea* mycelial plug was placed at the center of the plate, followed by incubation in the dark at 24 °C for 7 d. The colony diameter was then measured. In the control group, sterile water was used instead of the protein solution. For the treatment and control groups, two plates were inoculated per group. The experiment was carried out in triplicate and the entire experiment was repeated three times.

#### 2.4.4 Purified protein-induced hypersensitive response (HR) in tobacco leaves

After adjusting the protein concentration to 10 μM, tobacco leaves were infiltrated using the vacuum infiltration method as described above. The HR was observed after 3 d. Three tobacco leaves were inoculated per treatment, and the experiment was independently repeated three times.

### 2.5 Effect of GM001344-His on the induction of apple resistance to gray mold

#### 2.5.1 Apple treatment

Two 5-mm diameter wells (2 mm in depth) were created at opposing equatorial points of each apple using a cork borer. A 50 µL aliquot of the 10 µM purified protein solution was administered into each well. Following a 24-hour incubation period, 20 µL of a *Botrytis cinerea* spore suspension (1×10^6^ spores/mL) was introduced into the same wells for inoculation. For subsequent disease assessment, all treated apples were incubated in a growth chamber under conditions of 24 °C and 90% relative humidity for 8 d. Disease severity was rated on a scale of 0 to 4 based on the lesion diameter at the inoculation site: 0, no visible symptom; 1, lesion diameter ≤ 5 mm; 2, 5 mm < diameter ≤ 10 mm; 3, 10 mm < diameter ≤ 20 mm; 4, diameter > 20 mm (or with severe decay affecting over half of the fruit). Disease incidence (%) was calculated as the percentage of fruits showing symptoms relative to the total number of fruits treated. Disease index (DI) was calculated using the following formula: DI = [∑(Ni × Si)/(N × Smax)] × 100, where Ni is the number of fruits at severity level i, Si is the severity level (0, 1, 2, 3, 4), N is the total number of fruits assessed, and Smax is the highest severity level. Five apples were tested per treatment, and the experiment was performed in triplicate and repeated three times. Apples injected with sterile water and *B. cinerea* constituted control group.

#### 2.5.2 Apple fruit quality

Apples were sampled 6 d after treatment for evaluation of quality parameters (firmness, total soluble solids, reducing sugars, and titratable acidity), with three replicates of five fruits per group. Firmness (equatorial measurement) and total soluble solids were assessed according to Feng et al. (2025). Titratable acidity was determined by titrating a 10 g homogenate in 100 mL water with 0.1 mM NaOH to pH 8.3, expressed as % tartaric acid (Porat et al., 2000). Determination of ascorbic acid was performed by 2, 6-dichlorophenol indophenol titration. Fresh apple tissue was homogenized in oxalic acid on ice. Then the extract was filtered and the resulting filtrate was titrated with 2, 6-dichlorophenol indophenol. The results were expressed as mg per 100 g fresh weight.

### 2.6 Defense-related enzyme activities analysis

According to Feng et al. (2025), the activities of peroxidase (POD), and catalase (CAT), and phenylalanine ammonia-lyase (PAL) were assayed and expressed as U·kg^-1^.

### 2.7 Malondialdehyde content measurement

The extent of lipid peroxidation was determined by quantifying the levels of malondialdehyde (MDA) in the fruit tissue using the thiobarbituric acid assay (Senthilkumar et al., 2021), with results expressed as mmol MDA per kilogram of fresh weight.

### 2.8 Data statistics

The SPSS 27 program was used for data analysis. Experimental data were analyzed using one-way ANOVA followed by *post hoc* tests, with *P < 0.05* regarded as statistically significant. A minimum of three replicates were set for each treatment. GraphPad Prism 10 software was used to draw graphs.

## 3 Results

### 3.1 Prediction analysis of K1 genome secreted proteins and gene cloning

**Figure 1:**
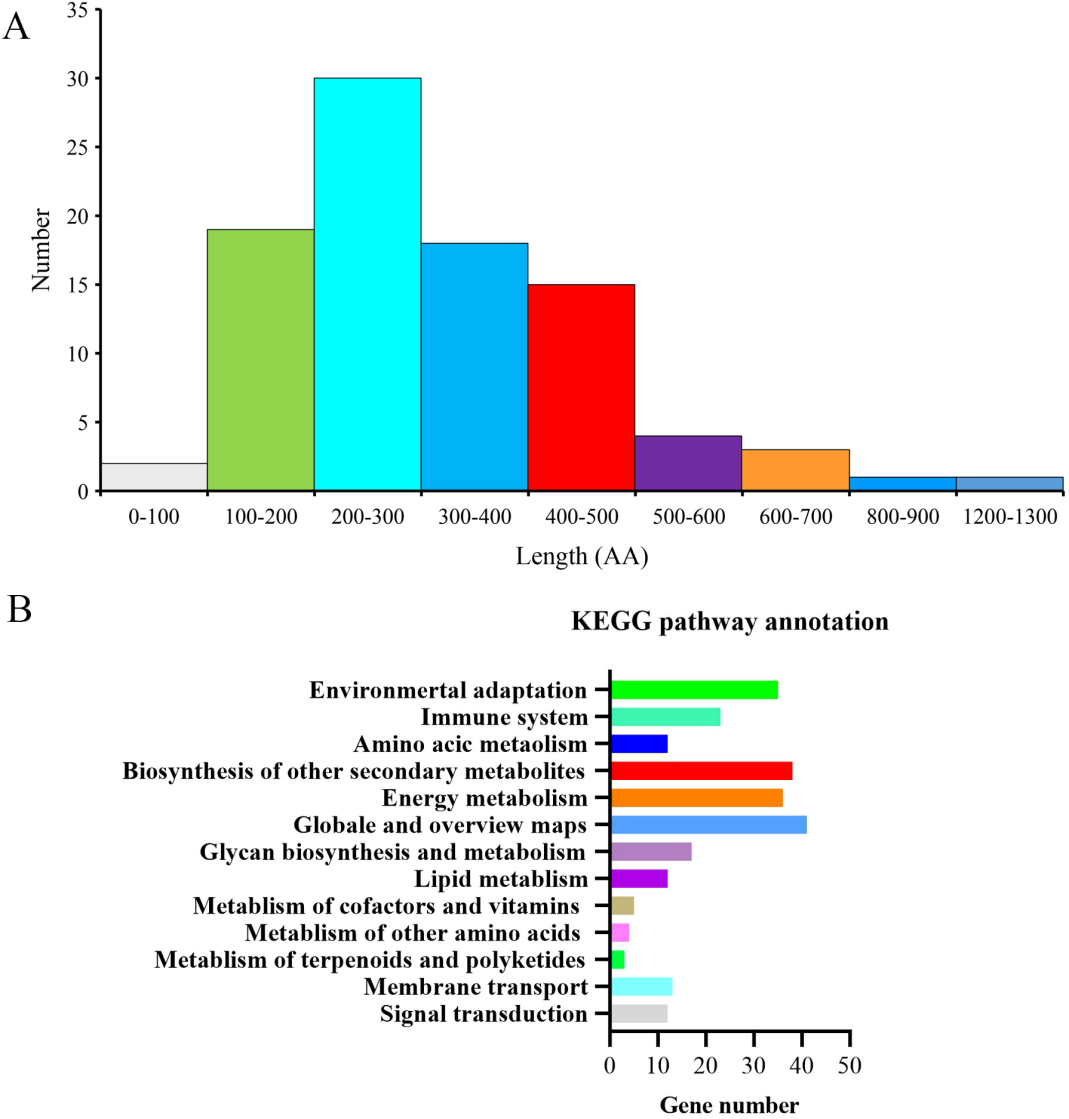
Analysis of 93 putative secretory proteins in the *Bacillus subtilis* K1 genome. **(A)** Distribution of amino acid sequence lengths. **(B)** KEGG pathway enrichment analysis.

Prediction of secreted proteins from the K1 genome identified a total of 93 candidate secreted proteins, which accounted for approximately 2.18% of the total predicted proteome (4263 proteins). And the secreted amino acid lengths were mostly between 100 and 500 aa, accounting for 87.23% (**Figure 1 A**). According to KEGG analysis, a significant majority of the identified proteins were primarily linked to pathways involved in environmental adaptation, biosynthesis of other secondary metabolites, and energy metabolism (**Figure 1B**).

Among the 93 predicted secreted proteins from *Bacillus* K1, GM001344 was selected for further study because it contained a signal peptide, lacked transmembrane domains, and had homologs known to be involved in bacterial physiology or plant–bacteria interactions. The candidate gene *GM001344* was cloned and sequenced. The sequence contained an open reading frame (ORF) of 642 bp, encoding a putative 213-amino-acid protein with a predicted molecular mass of 23.98 kDa. The protein was predicted to contain a 26-amino-acid signal peptide and had an isoelectric point (pI) of 5.45. GM001344 amino sequence exhibited no recognizable functional domains, and thus its biological function remained to be elucidated (**Supplementary Figure 1**).

### 3.2 Screening of elicitor protein by Agrobacterium-mediated transient expression

For transient expression mediated by *Agrobacterium*, both pvx-*GM001344* and Pvx- *GM001344*^ΔSP^ were successfully constructed (**Supplementary Figure 2**).

**Figure 2:**
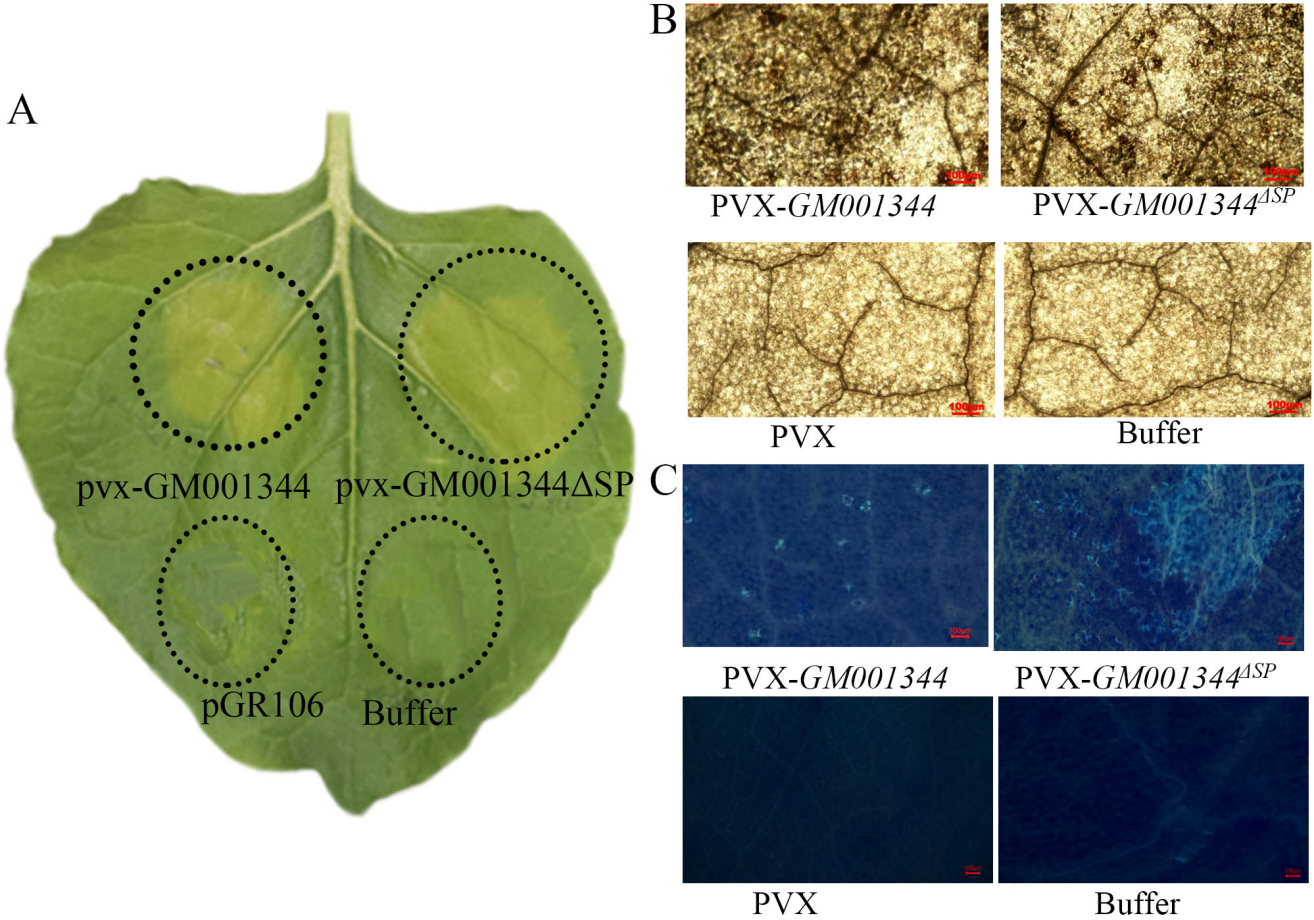
Transient expression of secretory protein GM001344 triggered a significant hypersensitive response in *Nicotiana benthamiana*. **(A)** Hypersensitive response (HR) phenotype. Infiltration zones showed the visible tissue response to (1) the full-length secretory protein (Full-length), (2) a mutant lacking the signal peptide (ΔSP), (3) an empty vector control (pGR106), and (4) infiltration buffer (Buffer). **(B)** ROS detection by DAB staining. **(C)** Callose deposition detected by aniline blue staining.

The HR phenotype was consistently observed in all infiltrated leaves. Over three independent biological replicates, a total of 9 leaves per treatment were infiltrated, and 100% (9/9) displayed a clear HR within 48 hours. A clear hypersensitive response (HR) was observed in all inoculated leaves at 48 h post-inoculation, characterized by yellowing at the inoculation site (**Figure 2A**). The accumulation of ROS in plants induced by transient expression of GM001344 was confirmed by DAB staining, which showed brown precipitate in the leaves (**Figure 2B**). Callose deposition was detected through aniline blue staining, which specifically binds to callose (**Figure 2C**).

### 3.3 Detection of HR and evaluation of antifungal efficacy of recombinant protein

**Figure 3:**
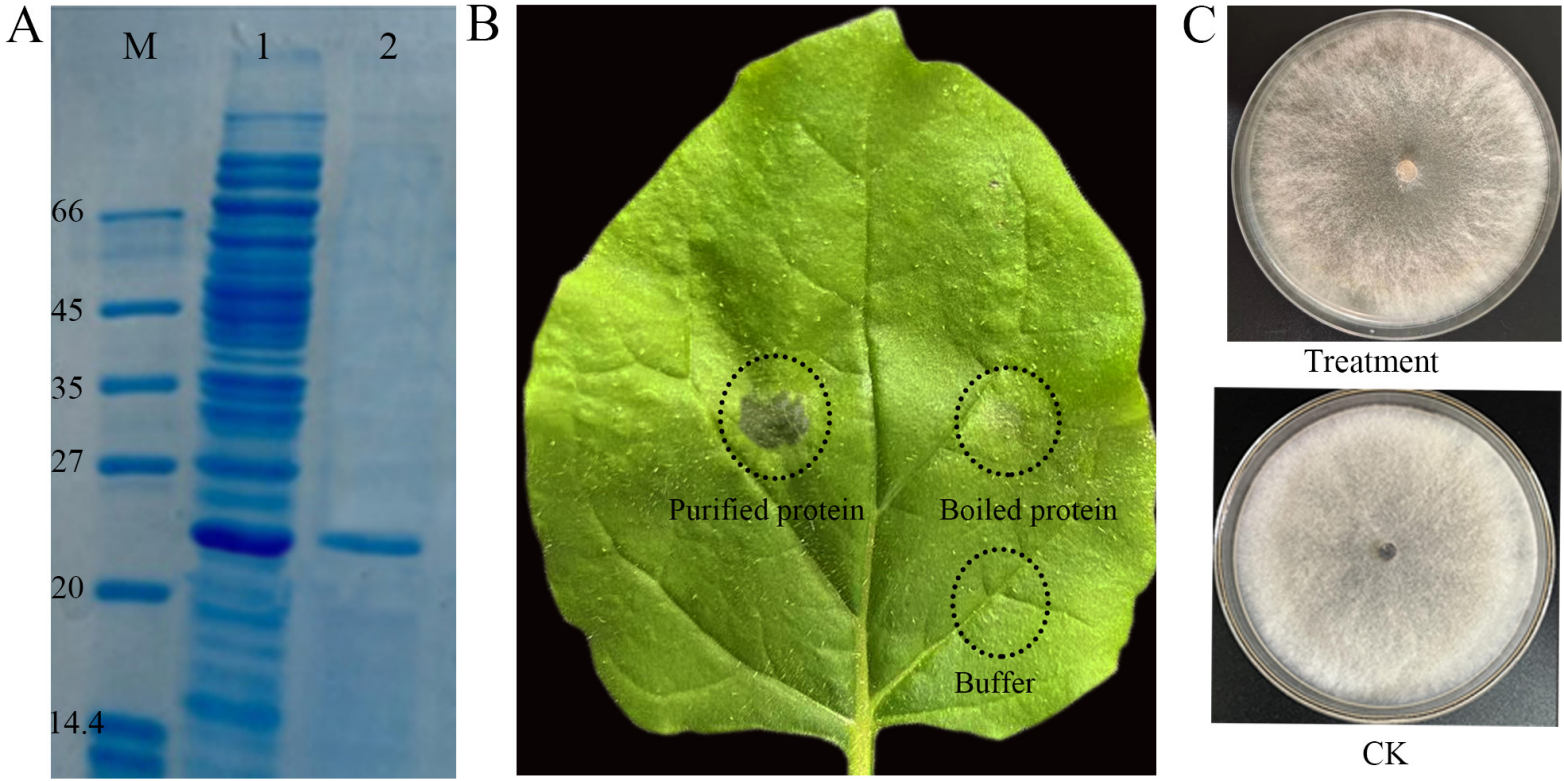
Expression, purification, and bioactivity assays of the secretory protein GM001344. **(A)** SDS-PAGE analysis of protein expression and purification. **(B)** Induction of HR on tobacco leaves by purified protein. Detached tobacco leaves were treated with: (1) the purified recombinant protein, (2) the boiled protein, and (3) buffer control. **(C)** Antifungal assay against *Botrytis cinerea*.

As shown in **Figure 3A**, the induced recombinant protein of *GM001344* gene had a size of approximately 24 kDa. After purification, it appeared as a single, clean band at the same molecular weight. When purified protein was inoculated into tobacco leaves, it induced a visible HR in all infiltrated tobacco leaves (n=9), whereas boiled protein or buffer alone failed to elicit such a reaction (**Figure 3B**). Moreover, the purified protein treatment showed no detectable inhibitory effect on the growth of *Botrytis cinerea* (**Figure 3C**).

### 3.4 Inhibitory effect of the purified protein GM001344-His on gray mold in apple fruit

**Figure 4:**
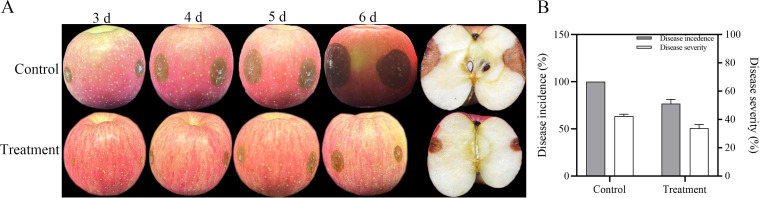
Application of the purified protein in apples against gray mold. **(A)** Disease progression. Apple fruits were treated with the purified protein or buffer 24 h prior to inoculation with a *B. cinerea* spore suspension. **(B)** Disease incidence and severity. Bars represent mean and error bars represent standard error of the mean.

In the control group, distinct fungal colonies were observed at the inoculation sites of fruits inoculated with *B. cinerea* on 3 d, and the lesions continued to expand thereafter. In contrast, the lesion areas on fruits in the treatment group were significantly smaller (**Figure 4A**). The incidence rate was 100% in the control group, compared to 63.5% in the treatment group, representing a 36.5% reduction. Regarding disease severity, the control group reached 76.75%, while the treatment group showed 38.43%. Calculations indicated that the treatment reduced the incidence rate and disease index by approximately 36.5% and 49.9%, respectively (**Figure 4 B**).

### 3.5 Apple fruit quality

The firmness of apples in treatment group was 12.84 N, showing a 3.38% increase relative to the control (12.42 N). The total soluble solids content in treated apples was 14.58%, which was 10.12% higher than that in the control group (13.24%). Additionally, a significant difference (*p* < 0.05) was noted between the treatment and control groups in terms of ascorbic acid content and titratable acidity (**Table 1**).

**Table 1:** Effects of GM001344-His protein treatment on apple quality parameters.

Treatments	Quality parameters			
	Firmness (N)	Total soluble solids (%)	Ascorbic acid (mg/100g)	Titratable acidity (%)
CK	12.42 ± 0.32b	13.24 ± 0.28b	0.17 ± 0.04b	3.54 ± 0.02b
Treatment	12.84 ± 0.13a	14.58 ± 0.47a	0.18 ± 0.02a	3.66 ± 0.03a

Mean values labeled with different letters are statistically different based on Duncan’s multiple range test (*P* < 0.05). Values were collected after 6 days of incubation at 24 °C. Treatment abbreviations: CK (control, inoculated with *B. cinerea* spores) and treatment (inoculated with GM001344-His protein followed by the pathogen).

### 3.6 Antioxidant enzyme activities and malondialdehyde content

**Figure 5:**
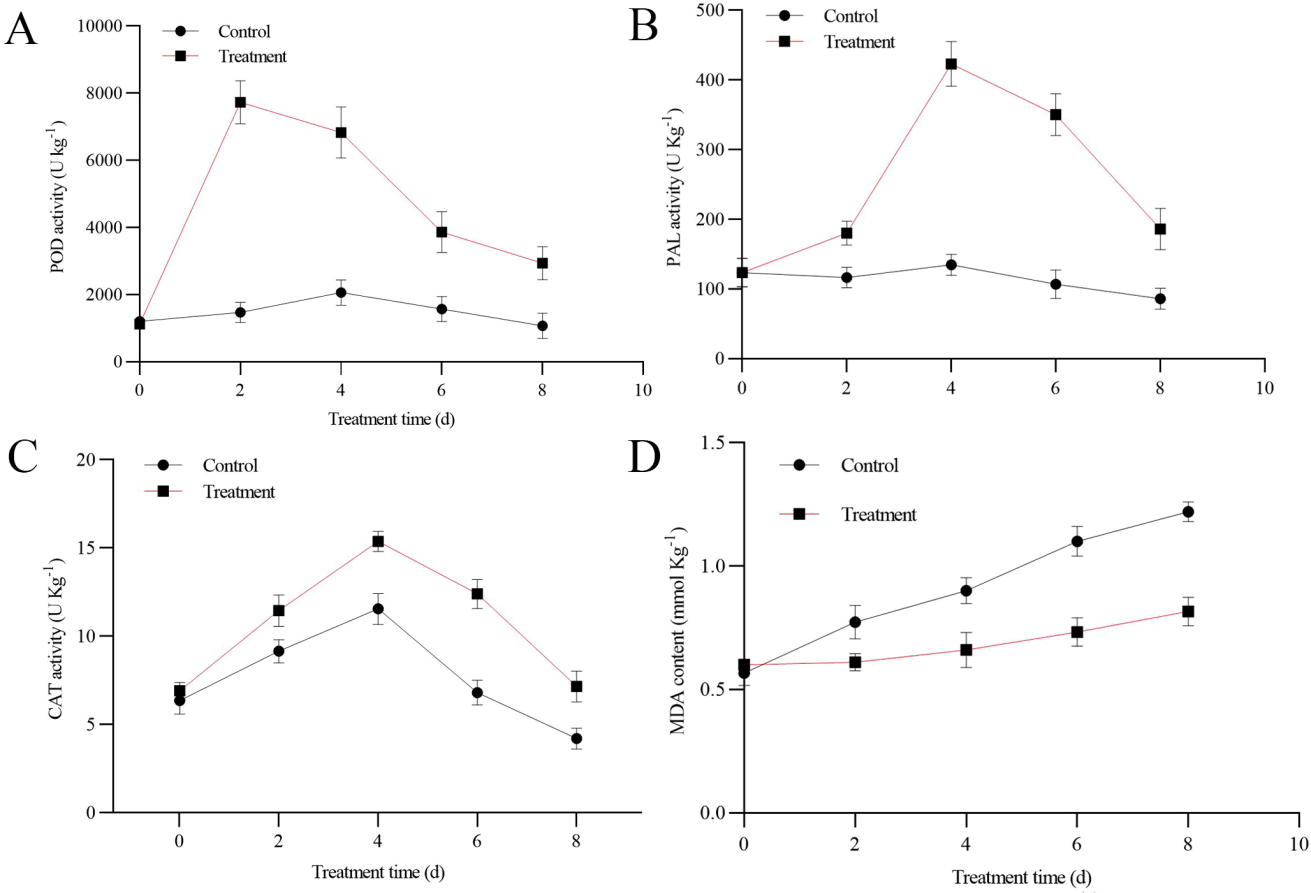
Effects of the purified protein on defense-related enzyme activities and malondialdehyde (MDA) content in apple fruits at 24 °C after treatment. Error bars represent the standard deviation (SD), indicating variability among biological replicates. **(A)** POD; **(B)** PAL; **(C)** CAT; **(D)** MDA content.

The activities of major antioxidant enzymes were assessed in apples from all experimental groups. POD activity in the treatment group exhibited a pattern of rapid increase to a peak at 2 d, followed by a gradual decrease, maintaining an overall significantly elevated level compared to the control (**Figure 5A**). PAL activity likewise demonstrated an initial increase, attaining a maximum at 4 d before declining, and was consistently and significantly higher in the treatment group (**Figure 5B**). A rise-and-fall trend was also observed for CAT activity, which peaked at 4 d in both groups. Importantly, the magnitude of activity was consistently and significantly greater in the treatment group (**Figure 5C**). A gradual increase in MDA content was observed over time in both the control and treatment groups. Despite this common upward trend, the MDA levels in the treatment group were consistently and significantly lower than those in the control group, indicating a protective effect of the treatment against membrane lipid peroxidation (**Figure 5D**). These results indicate that the protein elicited a specific response in the antioxidant defense system.

## 4 Discussion

Gray mold (*Botrytis cinerea*) is a primary cause of postharvest fruit decay, driving significant economic losses. Secreted proteins have emerged as promising agents in combating postharvest diseases of fruits and vegetables (Xu et al., 2024; Chen et al., 2023). In this study, we employed an integrated approach to identify and characterize the secreted protein GM001344 from the biocontrol strain *Bacillus subtilis* K1 that enhanced apple resistance to gray mold.

In this study, genomic analysis predicted a total of 93 exoprotein genes in *Bacillus* sp. K1, accounting for approximately 2.18% of all genes in the genome. The coding sequences of these genes mostly range between 58 and 1289 bp in length and are involved in various biological processes (**Figure 2**). These findings preliminarily indicated that these genes possessed certain functional diversity in secretion system, while also suggesting the presence of a considerable number of genes with unknown functions that warrant further investigation. The predicted exoproteins may play important roles in host interaction, environmental adaptation, and signal transduction. Similar functional characteristics have been reported for secreted proteins from other *Bacillus* species, such as *Bacillus velezensis*, suggesting that this may represent a conserved feature within the genus. For instance, a study on the *Bacillus subtilis* ULB16 demonstrated that its secretome not only contains highly active alkaline serine proteases but also exhibits notable biosurfactant properties, enabling dual applications in pollutant degradation and biofilm inhibition (Anand et al., 2025). The genomic analysis of the highly effective biocontrol strain *Bacillus velezensis* Y24 revealed a secretory system with considerable potential for producing diverse exoproteins including extracellular enzymes such as proteases and cellulases, as well as key antimicrobial lipopeptides (Li et al., 2025).This implies that the predicted exoproteins of the K1 strain may likewise constitute a multifunctional complex system, playing synergistic roles in environmental adaptation, substrate utilization, or microbial interactions.

GM001344 derived from the biocontrol agent *Bacillus* sp. K1, has been demonstrated in this study to trigger a robust early immune response in tobacco, characterized by reactive oxygen species (ROS) burst and callose deposition (**Figure 2**). This suggested that the protein likely functions as a microbe-associated molecular pattern (MAMP), which is recognized by plant pattern recognition receptors, thereby activating basal immunity. Such immune activation elicited by MAMPs from biocontrol agents can enhance the overall defensive capacity of the plant, providing a molecular explanation for the biocontrol functionality of *Bacillus* spp. In a parallel case, the protein elicitor AMEP412, derived from the probiotic *Bacillus subtilis*, effectively activated the rice immune system and enhanced resistance against rice blast disease (Qin et al., 2025). As another example, the protein elicitor PgTRX, derived from the antagonistic yeast *Pichia galeiformis*, effectively activated the immune system in citrus fruit and enhanced resistance against green mold disease (Zhu et al., 2026). Despite their known role in activating plant immunity through various elicitors, the precise mechanisms by which microbial biocontrol agents function demand deeper exploration (Zehra et al., 2021).

Purified protein GM001344-His showed no direct antimicrobial activity *in vitro* but elicited a typical hypersensitive response (HR) in tobacco (**Figure 3**). These results indicate that its primary function is not antimicrobial, but instead, it acts as an efficient elicitor of plant immunity. HR, a hallmark of elicitor-triggered immunity, suggests that the protein is specifically recognized by the plant immune system and activates robust defense signaling. Many known elicitors exhibit similar characteristics, lacking direct antimicrobial activity while being capable of inducing disease resistance. For example, purified protein elicitor (Cs08297) from *Ciboria shiraiana* could induce cell death in various plants and enhanced resistance (Zhang et al., 2024). Bioassays revealed that the plant elicitor LY5-24–2 lacked direct antimicrobial activity but effectively activated plant defense in *A. thaliana* through increased accumulation of lignin, cellulose, and pectin (Qi et al., 2022). Future work will focus on detailed structural and functional characterization of the protein GM001344 to elucidate the molecular mechanisms underlying its biological function.

In this study, apples treated with purified GM001344-His showed significantly lower disease incidence and severity than those in the control group under conditions of artificial inoculation with *Botrytis cinerea* spores. (**Figure 4**). Our findings added to the body of evidence that protein elicitors played a key role in mitigating fungal decay and enhancing the storage quality of fruit. For example, application of the protein elicitor PgTRX markedly suppressed both disease incidence and lesion diameter of green mold in citrus fruit (Zhu et al., 2026). The secreted protein PgPKC improved resistance against blue mold in citrus fruit by activating valine synthesis (Qiu et al., 2026). In conclusion, consistent with previous studies, our findings confirmed that the elicitor protein could significantly improve the postharvest preservation of apple fruit. Future work will therefore focus on evaluating the potential of GM001344-His to control natural decay in apples across a range of low-temperature storage conditions.

In the present study, the comparative stability of key quality parameters (firmness, TSS, AsA and TA) in the treatment group, relative to the rapid changes in the control, revealed a clear pattern of preservation (**Table 1**). This notable retardation in the rate of quality loss underscored GM001344-His-treatment efficacy. It implied that the intervention successfully mitigated the underlying physiological processes that lead to postharvest deterioration in apples. The stability of key quality metrics suggested a systemic modulation of fruit physiology, achieved through the downregulation of senescence-associated pathways. A phenomenon supported by studies on other elicitors in postharvest systems (Zhu et al., 2026; Qiu et al., 2026). Elicitor treatment with salicylic acid activated the phenylpropanoid pathway and promoted phenolic accumulation in jujube fruit, demonstrating its role in reprogramming postharvest physiology (Yuan et al., 2019). Future research should validate the efficacy of GM001344 across diverse apple cultivars and storage conditions and elucidate the signaling networks responsible for the observed metabolic changes.

Following pathogen infection, the accumulation of reactive oxygen species (ROS) is closely linked to early plant defense mechanisms. To mitigate the oxidative stress induced by ROS, plants activate their antioxidant defense systems. Treatment with GM001344-His was observed to enhance the activity of key antioxidant enzymes, including POD and CAT, in apple tissues (**Figure 5**). This finding aligned with multiple reports confirming the efficacy of secreted protein GM001344 in boosting enzymatic antioxidant capacity. Application of protein elicitor BvEP from *Bacillus velezensis* enhanced tomato resistance to *Botrytis cinerea* by upregulating the expression of plant defense-related genes and boosting the activities of superoxide dismutase (SOD) and peroxidase (POD) (Li et al., 2022). Furthermore, GM001344 treatment also increased the level of PAL higher than that in CK group. PAL is the rate-limiting and pivotal enzyme of the phenylpropanoid pathway and is responsible for synthesizing precursors for a range of resistance-related substances (Schwarze and Petersen, 2024; Gho et al., 2020). These secondary metabolites exhibit significant biological activities, and their accumulation is regarded as a key indicator of plant defense responses (Suo et al., 2025). Notably, the MDA content in the treatment group was significantly lower than that in the control group (**Figure 5D**). MDA is a well-established biomarker for lipid peroxidation of cellular membranes (Elbarbary and Mostafa, 2014; Yao et al., 2024). A progressive rise in fruit MDA content is commonly observed throughout storage, reflecting the accumulation of membrane peroxidation and oxidative injury (Liu et al., 2023). The present study found that application of GM001344 markedly suppressed the buildup of MDA. Accordingly, it can be inferred that this protein likely reduced the severity of gray mold infection by attenuating oxidative injury to cells and decelerating the breakdown of membrane lipids. Collectively, our data demonstrated that GM001344 likely functioned through the augmentation of antioxidant enzymes in apple fruit, leading to improved defense against gray mold.

## 5 Conclusion

In this study, a novel elicitor protein GM001344 derived from the biocontrol strain *Bacillus* K1 was identified. Following cloning and transient expression in *Agrobacterium*, the protein was shown to induce a typical hypersensitive response in tobacco. The protein was further expressed and purified using a prokaryotic expression system. Treatment of apple fruits with the purified protein effectively activated the immune response and enhanced resistance to *Botrytis cinerea*. Moreover, the treatment significantly increased the activity of defense-related enzymes in apples, while also maintaining favorable postharvest quality of the fruit. Collectively, these findings contribute to the development of sustainable biological strategies for preserving apples during postharvest storage.

## Data Availability

The datasets presented in this study can be found in online repositories. The names of the repository/repositories and accession number(s) can be found in the article/**Supplementary Material**.
