# Supplementary material for: Secreted proteins from Bacillus subtilis K1 induce apple resistance against gray mold
Source: Front Plant Sci. 2026 May 4;17:1825616. doi: 10.3389/fpls.2026.1825616 (PMC13180814; doi:10.3389/fpls.2026.1825616)
Supplement: Supplementary file 1 [file DataSheet1.pdf]

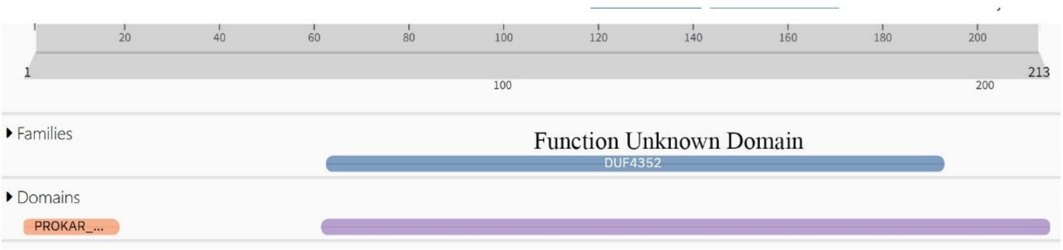

Fig.S1 Protein Domain Architecture Prediction of GM001344.

Prediction was performed on InterProScan (https://www.ebi.ac.uk/interpro/search/sequence/).

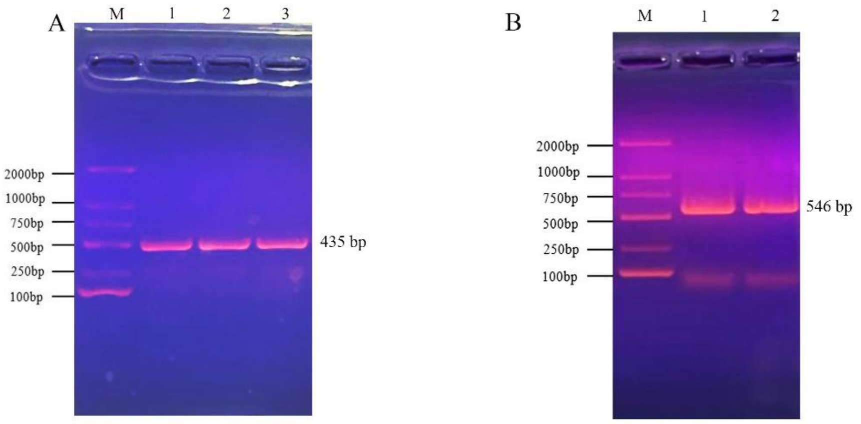

Fig. S2. Detection of recombinant plasmids by PCR.

(A) PVX-GM001344$^{\Delta SP}$, M: DNA marker; lanes 1–3: three independent clones. The expected sizes are 435 bp for GM001344$^{\Delta SP}$; (B) PVX-GM001344. M: DNA marker; lanes 1–2: two independent clones. The expected sizes are 546 bp for full length of GM001344.

Table S1 Primers used in this study.

Primer name	Sequence(5'to3')	Purpose
908-F	TTGTCAAAATGCGAGGAGGA	Amplification of the original gene sequence
908-R	TTATCGCATTTGTAAATCCTTTG	
1336-F	ATGAAGGCTTCATTGGTGTTATTC	
1336-R	TTAATAAGAGCCTGTGGTGGCCCA	
1344-F	ATGAAAAAAGTCTTATTACTATTAT	
1344-R	CTAATCTAACTTCACCAAGTAAT	
627F	ATGTTTTCATTGTCTGCGGGCAAA	

627R	TGATTTAGCTGAAAGCTCATCTTCTG	
1315F	ATGAAAAAGATTGTCCTTTCAAGTAT	
1315R	TTTTAATTTTCTTTTCCAGAGCC	
1467F	ATGCTCAAGAAAAAATGGATGGT	
1467R	GTCATCTATCTCCTGTTTGATAATGC	
1628F	ATGAAGAAAACATTTGTAAAAAAAG	
1628R	AGCCTGGGGATGAATCATAT	
1942F	ATGAGATTCACTAAGGTAGTTGGAT	
1942R	ATCATAAAACGTAACAGTTTTAGATT	
PVX-*1344*-F	GCACCAGCTAGCATCGATATGAAAAAAGTCTTATTACTATTAT (*Cla*I)	
PVX-$1344^{\Delta sp}$-F	GCACCAGCTAGCATCGATATGGTCAGAGAAGGAAAAACCGAA (*Cla*I)	Clone *GM001344* to PVX for expression in *N.benthamiana*
PVX-*1344*-R	CATTCATCGGCGGTCGACCTAATCTAACTTCACCAAGTAAT (*Sal*I)	
pET28a:1344-HIS-F	CAAATGGGTCGCGGATCCGAATTCATGGCGCCCCTTCACGACATATC(*EcoR*I)	Clone *GM001344* to pET28a for expression in *E.coli*
pET28a:1344-HIS-R	GTGCTCGAGTGCGGCCGCAAGCTTTCGCATTTGTAAATCCTTTGTG(*Hind*III)	
